# How Do Hospital Medical and Nursing Managers Perceive Work-Related Strain on Their Employees?

**DOI:** 10.3390/ijerph17134660

**Published:** 2020-06-28

**Authors:** Britta Worringer, Melanie Genrich, Andreas Müller, Florian Junne, Contributors of the SEEGEN Consortium, Peter Angerer

**Affiliations:** 1 Institute for Occupational, Social and Environmental Medicine, Centre for Health and Society, Medical Faculty, Düsseldorf University, 40225 Düsseldorf, Germany; peter.angerer@uni-duesseldorf.de; 2 Institute of Psychology, Work & Organizational Psychology, University of Duisburg-Essen, 45141 Essen, Germany; melanie.genrich@uni-due.de (M.G.); andreas_mueller@uni-due.de (A.M.); 3 Department of Psychosomatic Medicine and Psychotherapy, Medical University Hospital Tübingen, 72016 Tübingen, Germany; florian.junne@med.uni-tuebingen.de

**Keywords:** psychosocial stress, occupational health, healthcare, leadership, employee mental well-being, qualitative research

## Abstract

Health-oriented supportive leadership behavior is a key factor in reducing work stress and promoting health. Employees in the health sector are subject to a heavy workload, and it has been shown that 40% of them show permanent health problems. A supportive leadership behavior requires the manager’s awareness of the employees’ well-being. However, little is yet known about how medical and nursing managers perceive the well-being of their staff. To explore this issue, we conducted a total of 37 semi-standardized interviews with 37 chief physicians (CPs), senior physicians (SPs), and senior nurses (SNs) in one German hospital. The interviews were content-analyzed based on the definitions of strain of the ‘Federal Institute for Occupational Safety and Health’. Results show that hospital managers are aware of fatigue and further consequences such as deterioration of the team atmosphere, work ethics, treatment quality, and an increased feeling of injustice among employees. Most managers reported sick leaves as a result of psychosomatic complaints due to the permanent overstrain situation at work in the hospital. Results of this qualitative study are discussed in the light of health-oriented management relating to relevant stress models and to findings concerning staff shortages.

## 1. Introduction

Executives play a key role in shaping psychosocial working conditions by a health promoting leadership style [1]. In a recent meta-analysis, the following so-called styles of leadership behavior have shown to be beneficial to mental health of staff: transformational leadership, a high quality of relations-oriented and task-oriented leadership behavior, as well as a high quality of leader-follower interaction were positively associated with mental health [2,3], whereas destructive leadership was strongly negatively associated with mental health [4]. A common feature of the leadership behavior associated with mental health is an orientation towards a good relationship with the person that is led, by taking his or her opinion, needs, and well-being into account. Thus, for a health-oriented leadership in this sense and to offer social support, a leader must be able and willing to pay attention to the staff member’s subjective perspective.

Adverse working conditions (“work stress”) have a significant impact on the development of mental disorders such as depressive symptoms and adverse effects on emotion regulation capacity [5,6]. In addition, it can lead to an increased incidence of ischemic heart disease [7]. Working conditions in hospitals are characterized by a high level of work stress [8,9,10]. Compared to other occupational groups, employees in the health sector have the highest work intensity, the most frequent interruptions, and high emotional demands [11]. Consequently, 40% of employees in the health sector suffer from permanent health problems [12]. Burnout among doctors is a widespread and serious phenomenon [13]. Representative surveys found that almost every second (48.7%) surgical hospital physician in Germany [14] and almost one in three nurses [15] are affected by burnout. Therefore, work leaders’ social support is needed in hospitals to reduce work-related stress, to help employees cope with stress (i.e., stress is perceived as less of a burden), and to promote health since resources are built up as a result [16,17].

The training for medical and nursing staff is characterized by learning and practicing a lot of specialized knowledge, whereas the teaching of so-called soft skills and business or corporate concepts is missing [18]. Physicians acquire leadership mostly autodidactically, through observation and practice, regarding which it has been reported that most assistant physicians do not observe the most important situational leadership behaviors [19]. It is therefore not surprising that studies on management styles of hospital managers found that medical managers use a range of leadership styles [20] with two predominant styles: “dominance”, referring to control over tasks and the environment, leading others, and achieving goals, and “conscientiousness”, referring to working independently and a preference for working on tasks [21]. Both leadership styles focus on the task and on achieving goals rather than focusing on a good relationship between the manager and the employee. In 2010, the Center for Creative Leadership (CCL) found that the ability to lead employees and work in teams, along with self-awareness, was rated lowest of skills actually demonstrated by healthcare leaders [22]. Moreover, a systematic review found that leadership styles that focused on relationships were associated with higher nurses’ job satisfaction, whereas other studies showed that leadership styles that focused on tasks were associated with lower nurses’ job satisfaction [23]. High relationship-orientation among nurse managers was even associated with less sick leave among nurses [24].

Given the importance of a health-oriented leadership to reduce or buffer the detrimental effects of work stress in hospital work, little is known about how managers in nursing and medicine perceive the well-being of their staff. Recognizing the individual situations of staff members is a prerequisite for considering it a supportive relationship. Specifically, it is unclear to what extent hospital managers are aware of the work-related strain of their staff and if they perceive an influence of the strain on their staff’s health. For this reason, this qualitative study aims to investigate how senior hospital managers perceive work-related strain and strain consequences on their staff’s health.

## 2. Materials and Methods

### 2.1. Study Design

We conducted semi-standardized individual interviews with medical and nursing managers of one German hospital with two locations, belonging to a commercial hospital company. The bigger one has about 500 beds and employs about 700 physicians and nursing staff. The smaller one has around 350 beds and about 450 medical and nursing employees and was converted from a specialized clinic to an acute care facility.

In our study we present the key results of the interviews along the category system based on the definitions of strain of the ‘Federal Institute for Occupational Safety and Health’ [25,26]. Strain is defined as the direct effect of stressors on an individual, depending on his or her current and past circumstances, including individual coping strategies. Short-term strain can lead to positive or negative consequences. Positive consequences are to be expected if the level of stress is largely in line with the preconditions. If there are strong discrepancies (stress levels too high or too low), negative consequences are more likely to be expected (see [27]). Positive short-term consequences can be stimulation or activation, negative short-term consequences can be impairment (fatigue; fatigue-like conditions such as monotony, reduced alertness or saturation; stress symptoms). If the strain persists, long-term consequences (again positive or negative) may arise, like long-term positive consequences such as exercise, development of physical and mental abilities, well-being, health maintenance, or long-term negative consequences, such as general psychosomatic disorders and diseases (including digestive disorders, heart complaints, headaches), burnout, absenteeism, fluctuation, or early retirement. Consequently, based on the category system, these main categories are comprised: ‘negative short-term strain and mid-term strain consequences’, ‘positive short-term strain and mid-term strain consequences, ‘negative long-term strain consequences’, and ‘positive long-term strain consequences’ (Table 1).

### 2.2. Recruitment

Interviews were performed from April 2018 to July 2018. Participation was voluntary, but recommended by the hospital’s managing director, medical directors, and nursing service management. The participants were allowed to participate in the interviews during their working hours. The recruitment of chief physicians (CPs), senior physicians (SPs), and senior nurses (SNs) was done by (1) informing about the interview study at conferences of CPs and SNs, (2) calling attention to the study by e-mailing study information to the participants, and by (3) coordinating appointments by telephone to further inform about the study, partly via secretarial offices. The interview dates were personally arranged by telephone or e-mail with the participants and carried out ‘face to face’ at the participants’ workplace. Participants were informed about the data protection regulations and signed a consent declaration. It was almost always possible to conduct the interviews without interruptions (e.g., through emergency treatment). By approval of the participants, the conversations were recorded and lasted 45 min on average.

### 2.3. Design of Interview

We used interviews that emerged from a semi-standardized approach to the following research questions, that served to answer further questions [28,29]: ‘What do you think are the most important work stressors for your employees?’, ‘Which working conditions do you consider supportive and motivating for your employees?’, ‘To what extent do you see a connection between the stressors you have just mentioned and the mental health of your employees?’, ‘How do you describe your own role as a supervisor in relation to the mental health of your employees at work? ’, ‘How important is the mental health of employees in your hospital?’, ‘What opinions do your colleagues have on the subject?’, ‘What changes do you think can be implemented to relieve the strain on your employees in their day-to-day work?’, and ‘Do you personally see opportunities for yourself to maintain the ‘mental’ health of your employees at work and to reduce the stress you mentioned?’. The interviews lasted 45 min on average.

Interviews were conducted by three interviewers of the study team. After conduction of the first six interviews, there was another discussion to find out if it is necessary to make modifications, until no changes were necessary. The interviews were conducted until ‘theoretical saturation’ was achieved. Glaser and Strauss [30] defined these points of analysis at which ‘no additional data are being found whereby the (researcher) can develop properties of the category. As he sees similar instances over and over again, the researcher becomes empirically confident that a category is saturated. When one category is saturated, nothing remains but to go on to new groups for data on other categories, and attempt to saturate these categories also’ [30], p. 61, or [31].

### 2.4. Data Analysis

Interviews were recorded and transcribed by a specialized company and then analyzed by the study team using the conventional structuring content analysis [32,33,34] and by using the digital software MAXQDA 2018.1. We used the structuring content analysis, which pursues the goal of summarizing and systematizing the contents of the interviewees by theoretically derived dimensions in such a way that the results can be understood intersubjectively [35,36]. The creation and application of the category system was based on the deductive process using the definitions of the ‘Federal Institute for Occupational Safety and Health’ [25]. Definitions, anchor examples, and coding rules were defined for the categories. We tested the reliability in two steps: first, the formative reliability was tested; three members of the study team applied the coding guidelines based on three selected transcriptions and then checked and discussed the results concerning similarities and differences. The results were discussed with the large study team including the project leaders. Minor modifications to the coding guide were made. In the second step we tested the summative reliability. For this purpose, 2 × 4 transcribed interviews were coded by one researcher and counter-coded independently by another researcher. Based on the results, the interrater reliability was calculated. A Cohen’s kappa value of 0.82 indicated good reliability of our coding system [37]. In the first review of the transcriptions, the statements of the managers were assigned to the categories created. In the next step, we analyzed the statements within a category for a possible more in-depth systematization.

### 2.5. Sample

We interviewed 37 managers in total, including 14 CPs, 9 SPs, and 14 SNs working in different medical departments. We received no information from 6 SNs about the duration of employment in the current clinic, and 1 CP and 5 SNs lacked information on age. From the information available to us, the age range was from 34 to 60 years with a mean age of 51.9 years (CPs), 43.6 (SPs), and 47.9 (SNs). The average time of being employed in the current clinic was 5.4 years for CPs, 8.7 years for the SPs, and 23.4 years for SNs (Table 2).

## 3. Results

### 3.1. Positive Short-Term Strain and Mid-Term Strain Consequences

We identified only one statement of an SN, who said that employees who like to work and work overtime hours get financial support und are pleased with that. For this quote see Table 3, PST-1.

### 3.2. Negative Short-Term Strain and Mid-Term Strain Consequences

The most often mentioned negative strain was that managers reported that their employees appear exhausted (CP, two SNs, and one SP; NST-1), or unbalanced (one CP, one SP; NST-2), and that their employees have problems to relax after work (one CP, four SNs) due to the feeling of not having completed many tasks (one SP; NST-3-5). They also observed behavioral reactions and stated that their employees get insecure or anxious (one CP, two SNs; NST-6-7), or show aggressive or annoyed behavior (one CP, five SNs, one SP; NST-8-9) or emotional responses such as resignation, introversion, withdrawal (four CPs, one SN), frustration (one CP), pity (one CP; NST-10-11), or feelings of being overwhelmed by the variability of tasks (one SP; NST-12), which also influences the interaction between employees, e.g., by feelings of injustice (one CP, one SN, one SP), team atmosphere (four CPs), and work ethic (one CP; NST13-15). One manager reported that in overstrain situations, the employees try to pass the tasks on to the next higher level (one SP; NST-16), up to refusal to work (two CPs; NST-17). As further consequence of stress, deterioration of treatment quality (two CPs two SNs) was stated (NST-18-19), or even more careless mistakes, i.e., due to time-pressure (one CP, one SN, two SPs; NST-20). One SP also told us that an employee left the hospital as a reaction to a significant mistake he had made (NST-21). Selected quotes see Table 2, NST-1-21.

### 3.3. Positive Long-Term Strain Consequences

Six CPs reported they observe positive long-term strain consequences, such as a good atmosphere in the team (PLT-1-3), or that there are less downtimes (one CP; PLT-4), or that there is a low staff turnover (two CPs; PLT 5-6). Selected quotes see Table 4 PLT-1-6.

### 3.4. Negative Long-Term Strain Consequences

As the most frequent long-term strain consequence, 17 managers (five CPs, six SNs, six SPs) reported to perceive sickness leave (NLT-1-5). Some of them assumed that this could be a consequence of the body being on constant standby (one SN; NLT-6), which lowers resistance to illnesses (one CP; NLT-7), and then leads to illnesses (one CP; NLT-8). Matching this, managers reported to observe psychosomatic stress symptoms in their employees (one CP, one SP, three SNs; NLT-9-11), from sleep disturbances (one SP, two SNs; NLT-12-13), depressed mood, and burnout (one CP, five SNs; NLT-14-17) to workplace fear (one CP, two SNs; NLT-18-19) or anxiety due to being personally threatened (one CP; NLT-20). Another topic of long-term strain consequence reported by managers is that due to stress, conflicts and unrest in the team increase (two CPs, three SNs; NLT-21-23). One SP said that as a consequence of the greater anonymity in the care sector, quality and satisfaction of the individual colleagues also decrease (NLT-24), and another CP reported that an increased stress level leads to less identification with the department or with the employer and problems in team building (NLT-25), as well as to resigned behavior (one SP; NLT-26). Finally, it can even have the consequence that employees quit (two CPs one SN; NLT-27-29). For selected quotes see Table 4, NLT-1-29.

## 4. Discussion

The aim of this qualitative interview study was to investigate which short- and long-term strain consequences are perceived by hospital managers among their employees. By analyzing 37 interviews with 14 CPs, 9 SPs, and 14 SNs, we could identify a large number and variety of short- and long-term strain consequences perceived by managers, which we assigned to the category system according to the definitions of strain of the ‘Federal Institute for Occupational Safety and Health’ [25,26]. The investigated sample showed awareness for work-related strain of their employees and described them as very challenging, which provides the basis for a successful employee-oriented management style.

Overall, negative short- and long-term strain consequences were more frequently reported as compared to positive short- and long-term strain consequences. One participant mentioned financial compensation as short-term positive effect of the strain. Regarding positive long-term strain consequences, we found that managers perceive a good team atmosphere, less downtimes, and low staff turnover.

With respect to negative short-term strain consequences, managers reported to see their employees exhausted, unbalanced, frustrated, and resigned, and that this becomes evident in their behavior through insecurities, anxieties, or aggressive or annoyed behavior. Managers stated that they assumed that this tension worsened the team atmosphere and work ethic and could lead to an increased sense of injustice among employees with regard to the distribution of tasks, problems to switch off after work, and deterioration of treatment quality, or even careless mistakes.

### 4.1. Sickness Absence as a Result of Work Stress due to Staff Shortage

**Figure 1:**
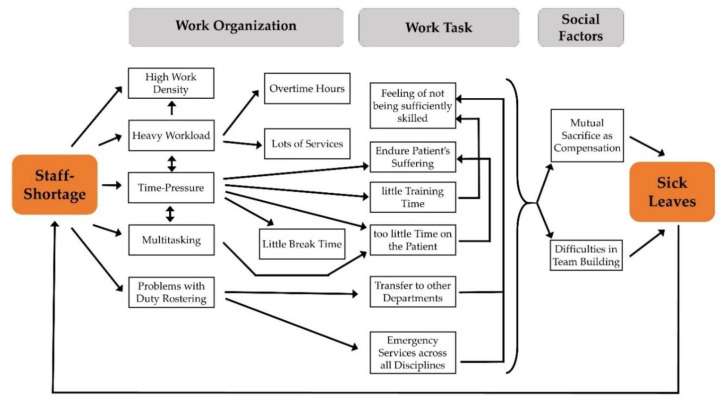
Graphical representation of the effect of staff shortage on further stressors relating to work organization, work task, and social factors (from [29]) extended by the mostly mentioned negative long-term strain consequence according to the hospital managers.

Sickness leaves were by far the most frequently reported negative long-term strain consequence. Managers reported to observe psychosomatic stress symptoms in their employees, such as sleep disturbances, depressed mood, burnout, and even workplace fear. Furthermore, it was hypothesized by participants that sickness leaves are a consequence of the body being in constant standby, which lowers resistance to illnesses. As an additional consequence, the managers reported that an increased stress level led to less identification with the department or with the employer. It further leads to disrupted team building and resigned behavior and can finally have the consequence that employees quit. In particular, the observation that most managers thought that sick leave was almost normal due to the stress situation indicates the seriousness of the stress situation in hospitals. Especially since another study indicates that staff shortages are an underlying factor that extremely exacerbates stressful situations in hospitals (see [29]), the observation that managers clearly attribute sick leave to work stress in the hospital may contribute to solutions for this unsolved problem. Thus, a downward spiral seems to have developed, which makes it difficult for hospital managers to counteract the immense burdens at the workplace by their own efforts (Figure 1).

Our results show that in our sample, the majority of managers see stress at work as a major reason for sick leave. The result is consistent with the meta-analysis of Duijts et al. [38], who found that a large proportion of sick leave could be attributed to psychosocial risk factors at work. Furthermore, Nieuwenhuijsen et al. [39] found on the basis of seven prospective studies that high work demands, low job control, low employee support, low supervisor support, low procedural fairness, low relationship fairness, and a high imbalance between effort and reward predict the incidence of stress-related disorders. Their findings are consistent with comparable results that found an association between psychosocial risk factors and the development of common mental disorders [40] and depressive disorders [5,41].

Sickness absence is a major problem in hospitals, and sickness absence research is a top priority. Reasons for sickness absence go beyond medical problems, as a complex mixture of legislation, processes, stakeholders, personality traits, and fitness levels of employees and circumstances that influence an individual’s decision to call in sick, stay at work or return to work [42,43,44]. A recent meta-analysis showed that fatigue, as described by the managers in this study, predicts sick leave [45]. However, several systematic reviews of conventional workplace interventions to reduce sick leave have yielded mixed results [46,47]. Thus, our findings may shed light on the importance of sufficient staffing in the hospital to reduce work stress, strain, and health consequences including sickness absence.

This view is corroborated by a recently published study [28] that showed that managers perceive decision latitude more at the individual and team level (e.g., through appraisal interviews, designing a fair work plan) and less at the organizational level. Various system-related reasons are cited by managers for not being able to create better working conditions by their own efforts through health-promoting work organization. In particular, for economic reasons in some cases, they feel externally determined by the top hospital management or the nursing service management and restricted in their work autonomy [28]. Since a health-related work design is very effective for economic success and for the quality of treatment, Genrich et al. [28] suggest that clear organizational norms and goals should be established in hospitals and that the health promotion of employees is an important organizational goal [48,49]. Sufficient staffing would be one prerequisite for making health promotion possible.

### 4.2. Limitations of the Study

Due to the voluntary participation of managers in the interview study, it can be assumed that we primarily reached those managers who already had a positive attitude towards the topic of employee mental health. We cannot therefore rule out a certain sampling bias. Furthermore, it cannot be ruled out that the participants in the interview situation showed socially desirable response behavior. Furthermore, we only interviewed the managers of one hospital, so that a generalization of the results may be limited. The interview guide used did not include direct questions about the managers’ perception of burdens among employees but asked the managers about the observed stressors and supportive factors for their employees. It is possible that asking directly for perceived burdens would have led to further answers. Moreover, questions like ‘What changes do you think can be implemented to relieve the strain on your employees in their day-to-day work?’ and ‘Do you personally see opportunities for yourself to maintain the ‘mental’ health of your employees at work and to reduce the stress you mentioned?’ may have biased the answers, because they suggest that there are mental stresses. Future research with more mixed or empirical methods is needed to corroborate the findings. Moreover, since we have examined the managers’ perspectives on the psychological burdens of their employees, we cannot make any statements about the actual burdens on employees. Future research could, for example, use mixed methods to investigate the actual psychological well-being of employees and the corresponding work characteristics using questionnaires or parallel interviews or focus groups.

## 5. Conclusions

This qualitative interview study indicates that hospital managers are aware of the strain of their employees. The hospital managers of the investigated sample are well aware of fatigue and the resulting behaviors and emotional states of their employees, which can have further consequences such as deterioration of the team atmosphere, work ethics, treatment quality, and an increased feeling of injustice among employees. Sick leave seems to be part of everyday business, as this was expressed by the majority of managers. The managers considered this to be the result of psychosomatic complaints due to the permanent overstrain situation at work in the hospital. Therefore, based on the perspective of experienced managers in the hospital, better staffing of nurses and physicians could help to reduce work stress, sick leave due to work stress, and could prevent the downward spiral. The managers’ awareness of the employees’ strain and of the relationship between adverse working conditions and poor employee health is a good prerequisite for health-oriented leadership behavior.

## Figures and Tables

**Table 1:** Category system for interview analysis.

Main Categories	Definition
Positive Short-term Strain and Mid-term Strain Consequences	The managers’ perception of positive reactions of employees to stressors, such as thoughts, feelings, physical reactions, behavior, and professional performance
Negative Short-term Strain and Mid-term Strain Consequences	The managers’ perception of negative reactions of employees to stressors, such as thoughts, feelings, physical reactions, behavior, and professional performance
Positive Long-term Strain Consequences	The managers’ perception of positive long-term reactions of employees to strain, such as psychosomatic disorders and diseases
Negative Long-term Strain Consequences	The managers’ perception of negative long-term reactions of employees to strain, such as psychosomatic disorders and diseases

**Table 2:** Demographic description of the sample.

	Chief Physicians	Senior Physicians	Senior Nurses
Female	2	2	9
Male	12	7	5
Total	14	9	14
Age range [years]	43–60	38–60	34–60
Mean age [years]	51.9	43.6	47.9
Mean number of years employed	5.4	8.7	23.4

**Table 3:** Selected quotes of the participants related to short- and mid-term strain consequences.

**Positive Short-Term Strain and Mid-Term Strain Consequences (PST)**
PST-1: There are still people who do, who like to come and work. This means not only these average planned services, but also when we really need and look for someone, they still come. Then of course we try to support them a little bit. And financially arrange it somehow. They are also in agreement with that. And also satisfied, actually. (SN 22)
**Negative Short-term Strain and Mid-term Strain Consequences (NST)**
NST-1: This, so to say this low staffing level, also leads to an I would say disproportionately high amount of on-call duties of individual employees. And this makes rest time almost impossible. And the employees appear extremely exhausted. (SP 33)
NST-2: But I think that many people would affirm the fact that you somehow feel unbalanced or other. (SP 35)
NST-3: That you really calm down, that is, in my opinion, such an important aspect. That you also learn how to switch off. A lot of nurses can’t do that either, they go home and they can’t get what happened at work out of their minds, it keeps them busy for hours. (SN 25). NST-4: You often call here from home, because you get some rest there and then you realize what you have forgotten, for example. (SN 28) NST-5: You go home in the evening with so many things in mind and feel like you’ve forgotten 20 things. (SP 34).
NST-6: Now we have to meet many, many, many demands and that makes some of the employees insecure and nervous. (SN 17) NST-7: My colleague quickly becomes restless in that case and is then briefly tied up, a bit grumpy towards the patients or towards me, when she feels a bit stressed. (SN 21)
NST-8: Yeah, so they either get indignant or they work even more slowly. (CP 2) NST-9: The colleagues are annoyed. ... That means that the colleagues simply become aggressive in tone at some point. Among each other. (SN 24)
NST-10: I think that a little bit of resignation resonates with everyone, because the moment someone fails, you have to fill this gap somehow. (SN 18) NST-11: So, the tasks are not carried out in the way you would have wanted, because the day has only 24 h and not 27 h to complete. And then, there is a really bad mood sometimes. …This means that from this side there is always a certain potential for conflict and this leads to a yes, resignation, an ‘I-don’t-care-mentality’, and then to a complete switch-off in the end. (CP 6)
NST-12: Yes, sometimes this is immediately reflected, that sometimes some employees reach their limits and say, I don’t know what to do first. (SP 40)
NST-13: But it is often the case that this feeling of injustice then becomes very strong. My burden is very great, and it is less great with the others. (SP 35). NST-14: Stress, yes, it does not necessarily help to maintain the team climate. And a bad team climate, I would say, messes up everyday life even more, and then there is a downward spiral. (CP 5) NST-15: But it is indeed the case that we sometimes resign ourselves to the clinical picture and are then frustrated that we have not managed to save human life. …Sometimes I see tears in the faces of the nurses or even doctors, for example when a course of treatment has not developed so well.… This is also noticeable not only visually, but you also feel that the mood is tilting. It becomes quiet. People are less open-minded towards each other. The many nurses and also doctors take this with them. And you notice a certain silence. And this silence always proofs that we were of course very dissatisfied and unhappy with the course of events. (SP 37)
NST-16: They are looking for a conversation with me, because then they often try to pass the tasks on to the next higher level and say, ‘Can you please decide that, I can’t do that. (SP 35)
NST-17: So, there are conflicts, it is rarely the case that there is rejection and open conflict and ‘I’m not doing that! Then I quit. (CP 13)
NST-18: Because just when they are really on their last legs, sometimes a little thing is forgotten, or they just can’t give 100%, different from what you are usually used to from your employees. (SN 26) NST-19: We can no longer give the medication, give tablets, care is omitted, only when really needed, and this is different from what we have learned in our training. (SN20)
NST-20: …and you can also tell by the mistakes. So now not treatment errors, but errors in the processes, uncertainties in the processes, which occur as a result. (CP 10)
NST-21: A colleague was doing a procedure on a patient. A complication occurred with this procedure. And this patient then became lifeless because of this measure, which was actually a very simple one, not such a highly dramatic one. He had to be resuscitated. And while we are carrying out the resuscitation measures on this patient, this colleague not only leaves the room, not only the intensive care unit, no, he leaves the hospital without saying a word, without signing out, but simply because he is dealing with this emotional situation, he has just been responsible for this event. (SP 37)

**Table 4:** Selected quotes of the participants related to long-term strain consequences.

**Positive Long-Term Strain Consequences (PLT)**
PLT-1: When people come up to me and tell me that they think it’s great what we have here. And what we do here. And that they feel good, and that they like working here. (ID 15) PLT-2: …at the moment I have the feeling that the mood in the team, at least with 90 percent, is very good. (ID 10) PLT-3: I have the impression that everything is working quite evenly in our department at the moment, and I would simply assume that they are all emotionally stable. (ID 12)
PLT-4: But the tendency, and I’m not alone now, is rather that rather, we have little downtime, so hardly anyone here is sick because of a little cold. (ID 11)
PLT-5: …many of them have already been there for a long time in these five years. And so, for once, the ones I took over, but also those who came after. They all like being here too. That is the case. Those who left had a hard time leaving. It was mostly for family reasons. (ID 2). PLT-6: We actually do not have such a high turnover of employees. (ID 9)
**Negative Long-term Strain Consequences (NLT)**
NLT-1: Well, I am sure that a relevant part of the sick days is like a call for help or dissatisfaction or maybe also caused by some kind of pressure. (CP 13) NLT-2: You could say they get sick if you push them too hard. Well, call in sick then. (CP 2) NLT-3: And work strain leads to reoccurring sick certificates, so that a very well-planned duty schedule has to be rescheduled all of a sudden. (SN 27) NLT-4: In the field of nursing, it must be said, there is understaffing, which favors overwork, sickness absence, so that the rat tail then, people always get sick. I believe that this is a problem of staffing, a personnel staffing problem. (SN38) NLT-5: We spend a large part of our lifetime here in the hospital or at work, and it is certainly the case that the employees are burdened by this daily work routine, by this stress and possibly also become mentally ill. The subject of burnout certainly also exists in the medical field and this hospital, the department not excluded, it is a topic. We somewhat notice this when employees are absent for longer periods of time because of somatic illnesses in fact, which are difficult to diagnose and record. For example, if I go into detail, myocarditis or myocarditis after an untreated flu is always something like that. This might sound strange now, but often employees who already are a bit older are absent for a long period of time. In the past years, in six years I have been here, we have had two employees who had been absent for six to eight weeks because of these complaints. This could actually be seen as some kind of new form of burnout or stress. (SP 40)
NLT-6: The load is so high that they can no longer relax at all. Because they’re required to step in constantly. You have to organize yourself. To jump in on weekends or days off, to come back from vacation, that certainly is a burden, because the body can no longer calm down. (SN 19)
NLT-7: The resistance to, let’s say, general illnesses is lowered when I basically have to work all the time in a state of exhaustion. (CP 11)
NLT-8: A permanent strain that cannot be overcome naturally makes you ill. Unfortunately, I have just experienced this with a long-time secretary. (CP 7)
NLT-9: And then even a slight headache can sometimes be like that, I will say now. Or a general feeling of unease. (CP 6) NLT-10: And since the workload is significantly higher and I then hear directly from the nursing staff, sometimes verbally, that there is dissatisfaction, relatively often, I also see nursing staff who present themselves with complaints here, where there is little organic correlation. At that point, you must already assume that there is a certain stress situation causing physical symptoms. (SP 36) NLT-11: That they simply also have physical complaints from time to time due to the mental strain. It’s all connected. Soul and body. And that they then also simply say they have headaches, one or two days in a row, but basically, it’s the great strain as an example. It happens. (SN 25)
NLT-12: There are colleagues who simply complain about sleep disorders. (SN 24). NLT-13: …she has got teeth grinding and also received a bite splint from the physician. She can barely relax at home either and still thinks, ‘Did I forget anything?’ She has trouble falling asleep and sometimes wakes up in the middle of the night, which she didn’t have before when she was working on another ward, with more staff and where things were not as concentrated on one person. (SN 21)
NLT-14: …there has been a real, yes disease state in the past years so that you could have equipped the whole hospital. For years, I have had more than thousand sick days every year in one single ward. People were indeed massively ill. On the one hand there were, typical illnesses for our professional life, slipped discs yes, this kind of issues. But there were also psychological stresses. Starting with burn-out. (SN 22) NLT-15: So, they may get physically sick faster, but I think they also become less resilient and that, I think, is always an expression of a yes, I would take it as exhaustion. You can take it to the point of exhaustion depression, which basically means that you lose motivation. (CP 11) NLT-16: So, it starts with you thinking, oh god, today again, probably it will come back, yes, I think so, it has something to do with the mood. Well, it is certainly not as good as it used to be. (SN29) NLT-17: Burnout. Yeah. And especially someone I would have never thought of. (SN 16)
NLT-18: And if this continues for days, the entire immune system collapses…don’t dare go back to the ward either. Some people also get scared of their jobs. (SN 20). NLT-19: You just come with a certain yes, also fear, I would say, no fear of work, but fear of what awaits me. Can I manage the work at all? (SN 24)
NLT-20: In individual situations, this can of course lead to such a heavy burden for the affected person that he or she says, ‘I don’t feel well there, I’m afraid to do emergency services. And then they withdraw. …So, there is a very clear threat to employees, who can then also develop personal fear. (CP 9)
NLT-21: We have had a time where the conversational tone among each other has also been very bad. (CP 10) NLT-22: And I think this uncertainty also leads to unrest in the team and to many people looking at each other, comparing each other, who does more of this or that.’ …’ you already notice this in rush hours, that people then go at each other’s throats for a little something, and you think they would not have done that at all under normal circumstances. (SN 17) NLT-23: They feel totally overloaded. And they officially say so. Of course, this also leads to stress situations in the team, with the same people being affected over and over again, because the stronger people naturally assert themselves and they simply don’t come. (SN 22)
NLT-24: And in nursing, the areas are being expanded, so that there is actually more and more anonymity, which has the advantage, from a business point of view, that people are interchangeable. And if somebody gets sick, I can very quickly swap with someone else. In my opinion, the quality and satisfaction of the individual colleagues also decreases. Simply because this appreciation is missing in my everyday life. (SP 36)
NLT-25: The connection then with the company, the connection with your employer or your clinic, which was perhaps always the case in the past, does certainly not exist today. (CP 6)
NLT-26: Because of course there are also certain colleagues of mine with whom one perhaps cannot discuss such things constructively. But even a little bit of resignation yes, so when they do, I often have the impression that the assistants simply have to go out into the street a bit, to put it in a figurative way, and nobody complains. There are no complaints. (SP 38)
NLT-27: The market works in such a way that doctors in the medical sector are then able to change employers. You can also see that if the fluctuation in the individual departments is high, then you can see that there might also be a need for action in these areas. (CP 2) NLT-28: One employee in December certainly left because she could not handle this workload. (CP 10) NLT-29: There used to be a few of them, but they left. At some point they drew a line under it and said they had to work somewhere else. (SN 17)
